# Optimization of Electron Beam Welding Joint of Water-Cooled Ceramic Breeder Blanket

**DOI:** 10.3390/ma14123405

**Published:** 2021-06-19

**Authors:** Yong Zhang, Jiefeng Wu, Zhihong Liu, Songlin Liu, Mingzhun Lei, Muhammad Atif, Zhenfei Liu, Xu Shen, Jianguo Ma

**Affiliations:** 1 Institute of Plasma Physics, Hefei Institutes of Physical Science, Chinese Academy of Sciences, Hefei 230031, China; zhang.yong@ipp.ac.cn (Y.Z.); jfw@ipp.ac.cn (J.W.); zhliu@ipp.an.cn (Z.L.); slliu@ipp.ac.cn (S.L.); leimz@ipp.ac.cn (M.L.); zhenfei.liu@ipp.ac.cn (Z.L.); shenxu@ipp.cas.cn (X.S.); 2 Science Island Branch, University of Science and Technology of China, Hefei 230026, China; 3 CAS Key Laboratory of Soft Matter Chemistry, Department of Polymer Science and Engineering, University of Science and Technology of China, Hefei 230026, China; atifarain@mail.ustc.edu.cn; 4 Anhui Province Key Laboratory of Special Welding Technology, Institute of Plasma Physics, Huainan 232000, China

**Keywords:** reduced activation ferrite/martensite steels, electron beam welding, metallographic structure, mechanical properties

## Abstract

The water-cooled ceramic breeder (WCCB) blanket is a component of the China Fusion Engineering Test Reactor (CFETR). The Reduced Activation Ferrite/Martensite (RAFM) steels are the preferred structural materials for WCCB blanket. As a kind of RAFM steels, China low activation martensitic (CLAM) steel was welded by electron beam welding (EBW), and then quenched-tempered treatment was carried out. The results show that at the welding state, the welding seam was composed of large martensite and δ ferrite and the organization of the heat-affected zone (HAZ) was changed slightly with the different heat input. Moreover, the hardness of welded joints was higher than that of base material (BM), but the impact toughness was very low. After quenched-tempered treatment, the δ ferrite in the weld was eliminated, the residual stress of the test plate decreased as a whole, and the mechanical properties were improved significantly.

## 1. Introduction

**Figure 1:**
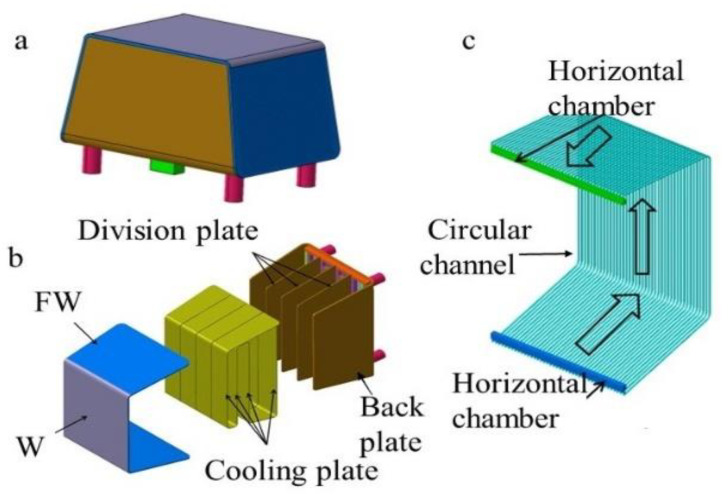
The structure of WCCB blanket. (**a**) WCCB blanket (**b**) internal structure of WCCB blanket (**c**) internal structure of FW. (Reprinted with permission from ref. [12] © (2021) Elsevier).

Nuclear fusion energy is generally considered to be the main energy for human development in the future [1,2]. As one of the core components of nuclear fusion, the water-cooled ceramic breeder (WCCB) blanket produced by Institute of Plasma Physics, in Hefei, China can shield neutron radiation and produce tritium during the operation of fusion reactor. Figure 1a shows the WCCB blanket of the China fusion engineering test reactor (CFETR) produced by Institute of Plasma Physics, in Hefei, China. As far as the structure–material of the WCCB blanket is concerned, it needs to support the stress caused by complex structure of WCCB blanket, needs to take a long time, and high doses of neutron irradiation [3], as well as high pressure and temperature liquid-lithium lead corrosion [4], etc. These factors determine that the structure–material must have excellent physical and mechanical properties. Reduced activation ferrite/martensite steel (RAFM steel) has excellent thermal physical and mechanical properties such as high radiation swelling resistance, thermal conductivity and low coefficient of linear expansion. It is generally considered as the preferred structural material for the test blanket module (TBM) of fusion test reactor in the world. Many countries in the world have carried out a lot of work in the development and characterization of these materials, such as China low-activation martensitic (CLAM) steel produced by Institute of metals, in Shenyang, China [5], Japan’s F82H [6,7], the European Union’s EUROFER97 [8] and Rusfer EK-181 [9]. Due to the complex structure of WCCB blanket and extremely harsh service conditions, the quality of welding and the selection of heat treatment processes play a decisive role in the feasibility and safety of the fusion reactor operation [10].

The first wall (FW) (Figure 1b) is one of the components of the WCCB blanket. CLAM steel was selected as the structural material of FW. It is difficult to control welding deformation because the FW contains dense flow channels (Figure 1c). Due to the advantages of small welding deformation and high efficiency, electron beam welding (EBW) was used as the welding method of FW. Some scholars have studied the EBW joint of RAFM steel. For example, Hu Jie, et al. [5] have studied the EBW joint of RAFM steel, and found that the properties of the joint after quenching and tempering treatment are better than those only after tempering treatment. Jiang Zhizhong, et al. [11] found that the hardness of the welded joint is greater than that of the base metal, and after heat treatment, the hardness decreases and the joint performance improves as a whole. However, these studies have not carried out in-depth analysis of EBW joint stress under different states.

**Figure 2:**
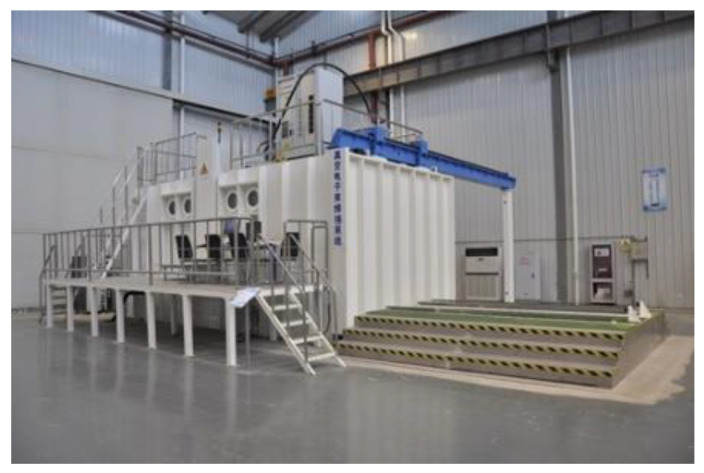
The equipment of EBW. (Reprinted with permission from ref. [13] © (2019) Elsevier).

The actual thickness of the FW-EBW joint is 23 mm. Therefore, we studied the EBW process and heat treatment process of 23 mm thick CLAM steel. The EBW equipment is shown in Figure 2. Tensile, impact and transverse bending tests were carried out before and after heat treatment. All heat treatment processes were carried out under atmospheric pressure. The corresponding changes of microstructure were studied, and the relationship between microstructure and mechanical properties was revealed.

## 2. Materials and Methods

**Figure 3:**
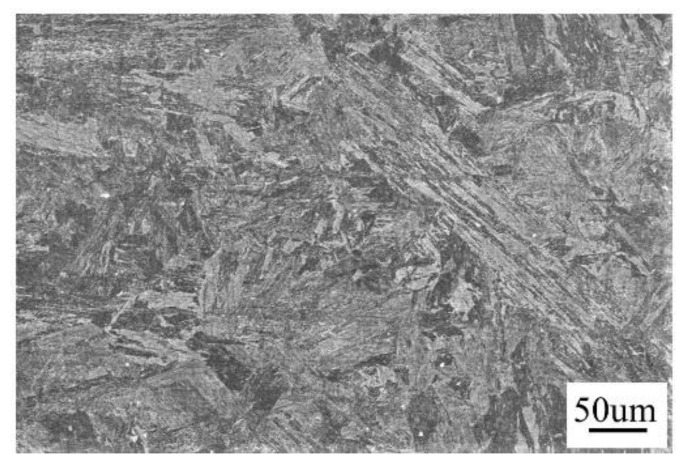
Microstructure of CLAM showing tempered martensite.

The as-received steel was at normalized and tempered state. The parameters of normalizing and tempering are: keeping for 40 min at 980 °C, air cooling; maintained for 40 min at 760 °C, and air cooling to room temperature, respectively. The chemical composition was measured by direct reading of the spectrometer produced by gangyannake Testing Technology Co., Ltd. in Shanghai, China (Table 1) and having the microstructure as tempered martensite (Figure 3). 

**Figure 4:**
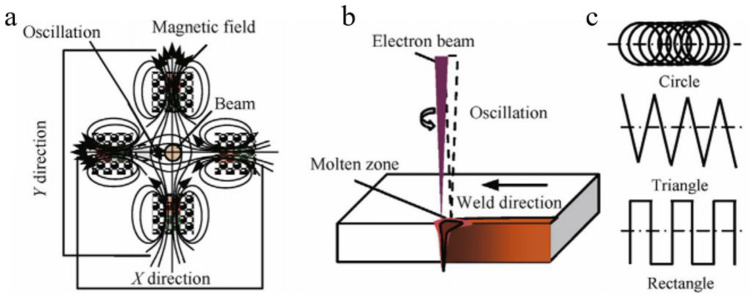
Sketch of EBW with beam oscillation. (**a**) Oscillation with electromagnetic fields. (**b**) Beam oscillating welding. (**c**) Wave function. (Reprinted with permission from ref. [13,14] © (2019) Elsevier, (2019) Elsevier).

We have prepared six steel plates and butt welded them separately. The size of each plate is 300 mm × 100 mm × 23 mm. The welding joint is composed of two pieces of steel plate along the length direction. The surface scale on the steel plate was removed by mechanical polishing and then swabbed clean by anhydrous ethanol. Before EBW, the steel plates to be welded were restrained. The equipment of EBW is ZD150-60C CV66M type vacuum electron beam welding machine produced by Sea-Sun-Tech, Trappenkamp, Germany (Figure 2), with vacuum degree of work is about 1.7 × 10^−4^ mbar, and the acceleration voltage is 150 kV. In order to obtain high-quality welding seams, scanning deflection of the electron beam was used in the test (Figure 4).

Scanning deflection can avoid weld defects by reducing root bounce and stirring the weld [15]. In order to obtain fully penetrable welded joints, the welding parameters were optimized on four 23-mm-thick CLAM steel plates. The sizes of the plates were 300 mm × 200 mm × 23 mm, and the parameters before and after optimization are shown in Table 2.

X-ray beam was used to check the quality of the welding joint. The brand of X-ray testing equipment is XXG-2505. The voltage we used was 20 kV, the current was 5 mA, and the focal length was 600 mm. The quality of the image was verified by a wire image quality indicator (IQI). The minimum IQI value was W12 (0.25 mm) according to the standards (ISO17636). Here, any kind of porosity inclusion and defects were not found. The residual stress of two welded test plates before and after heat treatment was analyzed by the blind hole method. According to the distribution regulation of residual stress, dense test points were arranged in the area near the weld, while loose test points were arranged in the area far away from the weld (affected by the weld reinforcement; the residual stress at the weld cannot be measured; thus, we have only measured the residual stress on the base metal (BM)).

**Figure 5:**
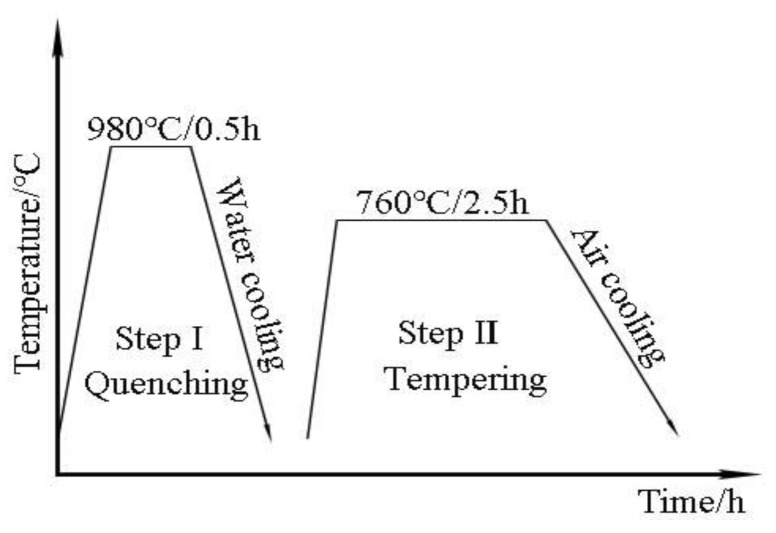
Heat treatment process.

The distribution of residual stress on both sides of the weld is symmetrical, so we only studied the residual stress distribution on one side of the welds. The other board was divided into two parts from the length direction, and one piece was treated with quenching and tempering. The parameters of quenching and tempering are keeping for 30 min at 980 °C water quenching, again keeping for 150 min at 760 °C, and air cooling to room temperature, respectively [16] (Figure 5). The samples for metallographic analysis were prepared according to the standard of ISO 17639: 2013 [17], followed by corroding with aqua regia (75 wt % HCL + 25 wt % HNO_3_). The microstructure of etched samples was studied by DHV-1000Z optical microscope and SIGMA300 field emission scanning electron microscope (FESEM) produced by Carl Zeiss AG. in Jena, Germany. According to GB/T 2654-2008/ISO 9015-1: 2001 standard, the microhardness of welding joints before and after heat treatment was measured by DHV-1000Z Vickers microhardness tester, with a loading load of 4.9 N and a hold time of 15 s. The impact, tensile and lateral bending tests were carried out to study mechanical properties of base material (BM), and welding joints before and after the heat treatment. According to GB/T 2650-2008/ISO 9016: 2012 standard, impact samples adopted a V-shaped notch, and the notch was opened on the side. The size of samples was 55 mm × 10 mm × 10 mm; all of them were divided into three groups, and each group had three samples. The impact testing machine used was a Jbw-300b microcomputer semi-automatic impact testing machine/170615. According to GB/T 2651-2008/ISO 4136:2012 standard, the tensile testing samples were divided into three groups; the tensile test machine used was a WA-600A/electro-hydraulic universal testing machine/060201, according to GB/T2653-2008/ISO 5173: 2009 standard. Moreover, the bending test samples were divided into two groups; the bending testing machine used was a WA-600a electro-hydraulic universal testing machine/060201.

## 3. Analytic Procedure

### 3.1. Residual Stress

**Figure 6:**
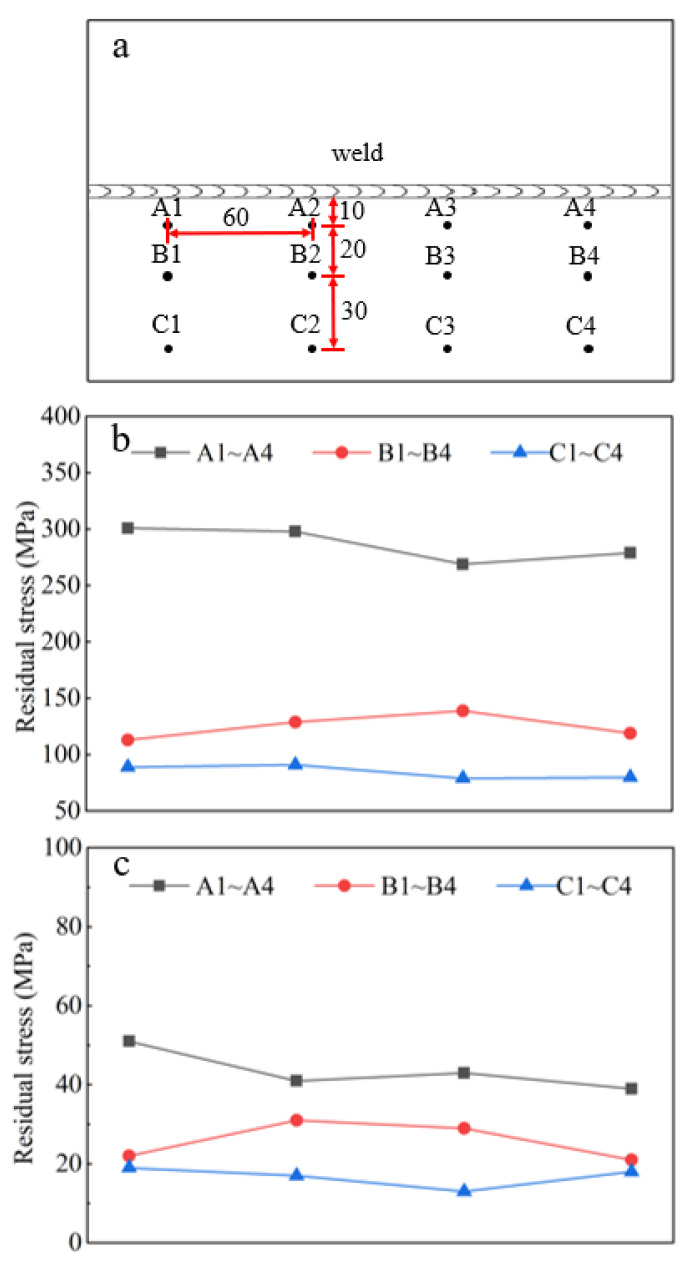
Diagram of residual stress. (**a**) Strain gauge distribution, (**b**) in welded state, and (**c**) after quenching and tempering.

The residual stress distribution of the heat-affected zone (HAZ) and BM before and after quenching and tempering was measured by the blind hole method. As the residual stress on both sides of the weld has the characteristic of symmetrical distribution, we only tested one side (there is reinforcement at the welds, which cannot be measured, and removing the reinforcement will affect the stress distribution) (Figure 6). Figure 6a shows the distribution of the strain gauge when measuring the residual stress on the test plates. The transverse distance between the A1 to A4, B1 to B4, and C1 to C4 points and weld was 10 cm, 30 cm, and 60 cm, respectively, and the longitudinal distance of these points was 60 cm. Figure 6b,c show the stress distribution before and after heat treatment. In the welding state, the maximum residual stress is about 300 MPa. The farther away from the weld, the smaller the residual stress is, and its minimum value is 79 MPa (Figure 6b). After heat treatment, the residual stress decreases as a whole, the maximum and minimum values are about 51 MPa and 13 MPa, respectively (Figure 6c).

### 3.2. Mechanical Properties

#### 3.2.1. Impacting Test

**Figure 7:**
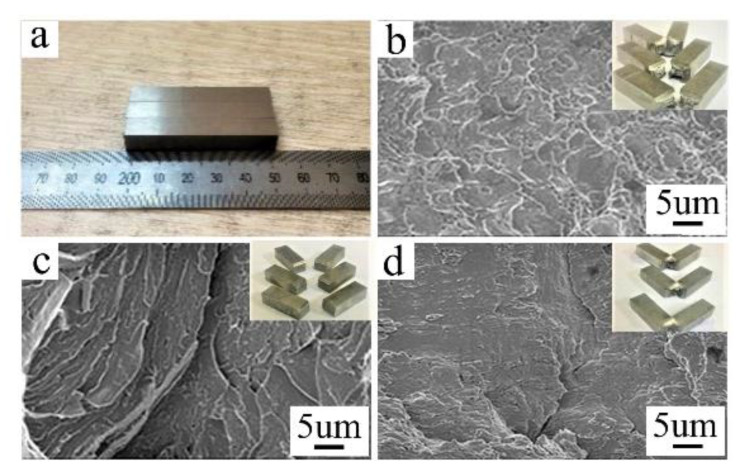
Impact test samples; (**a**) samples before testing, (**b**) fracture morphology of CLAM steels, (**c**) fracture morphology of EBW joints in welding state, (**d**) fracture morphology of EBW joints in quenching and tempering state.

Figure 7a shows the impact test samples. In Figure 7b, d, there are obvious macroscopic plastic deformations near the fracture surface, the fracture surfaces have serpentine slip patterns and a rippled appearance, respectively, which are typical of plastic fracture. Under the welding condition, there is no obvious plastic deformation near the fractures. The fracture morphologies of the welded joints are even, with radial tearing prism; this is a typical brittle fracture (Figure 7c).

**Figure 8:**
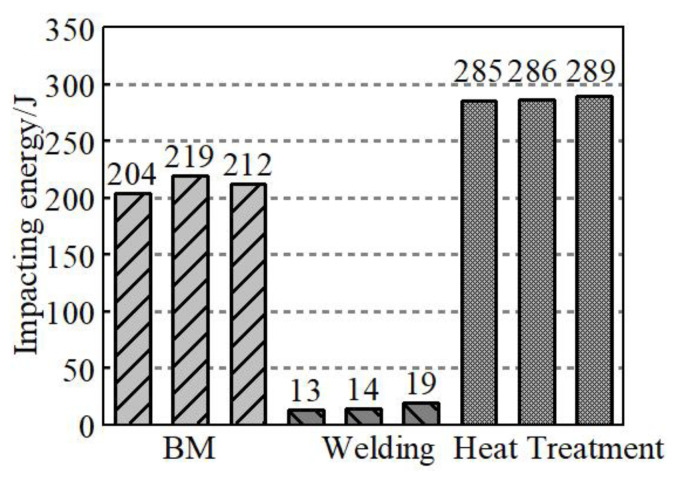
Impact energy of samples.

Figure 8 represents the room temperature testing results of BM, welding status, quenched and tempered samples. The test results show that in the state of the welding (room temperature 25 °C), the impact toughness value is extremely low, which is only about 7% of the BM, and 5% of the quenching and tempering state. On the one hand, this phenomenon is related to the precipitation of chromium and other elements in ferrite and carbide at high temperature. On the other hand, the existence of the quenching structure and δ ferrite structure itself may be the main reason for welding brittle fracture. The low-impact absorption energy of welded joints clearly indicates that the welded joint needs post-weld heat treatment. However, one-step tempering cannot significantly improve the impact energy of the welded joints [10,18]. The impact toughness of welding joints was improved significantly after quenching and tempering, and it is equivalent to 135% of the impact absorption energy of BM. The impact toughness of welded, quenched and tempered joints should be analyzed in combination with the microstructure of the joints.

#### 3.2.2. Tensile Test

**Figure 9:**
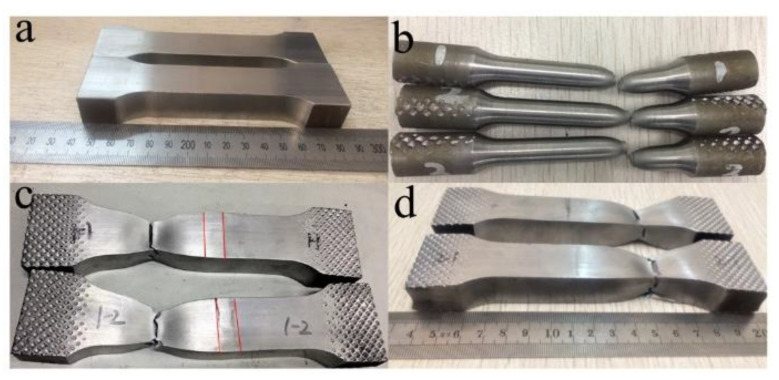
Tensile test samples; (**a**) samples before tensile test, (**b**) BM samples after test, (**c**) samples after test under welding condition, and (**d**) samples after test under quenching and tempering condition.

Figure 9 shows the tensile test samples. Figure 9a shows the samples before tensile test, Figure 9b contains the BM samples after test, Figure 9c shows the samples under welding conditions after test, and Figure 9d represents the samples under quenching and tempering condition after test. After the tensile test, the samples in the welding state and quenched and tempered state fracture on BM, which proves that the microstructure strength of welded joint is greater than BM. The difference is that in the welding state, the microstructure of the weld is obviously prominent after the test, showing the characteristics of high hardness.

#### 3.2.3. Lateral Bending Test

**Figure 10:**
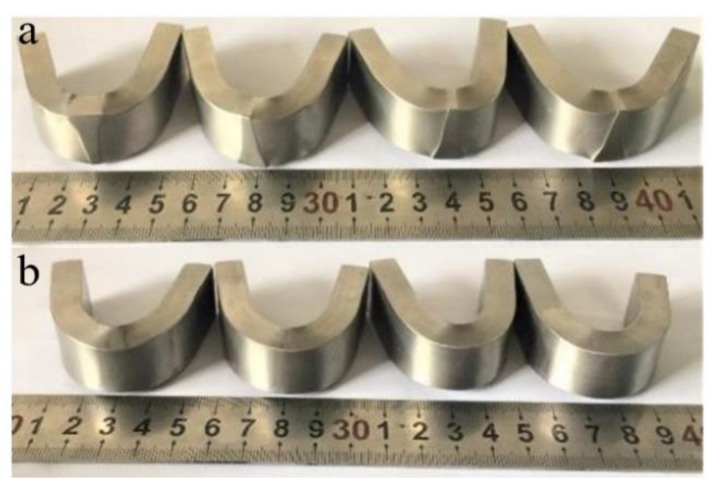
Bending test samples; (**a**) samples after test under welding condition, and (**b**) samples after test under quenching and tempering condition.

After the bending test, the microstructures of the welded joints were prominent under the welding condition, showing that the hardness is greater than that of the BM (Figure 10a). This phenomenon did not appear in the quenched and tempered specimens (Figure 10b). In both cases, no cracks appeared in the weld after the test, which indicates that the weld microstructures are well combined with the BM.

### 3.3. Microstructure Analysis

#### 3.3.1. Microstructure Distribution of Welded Joint

**Figure 11:**
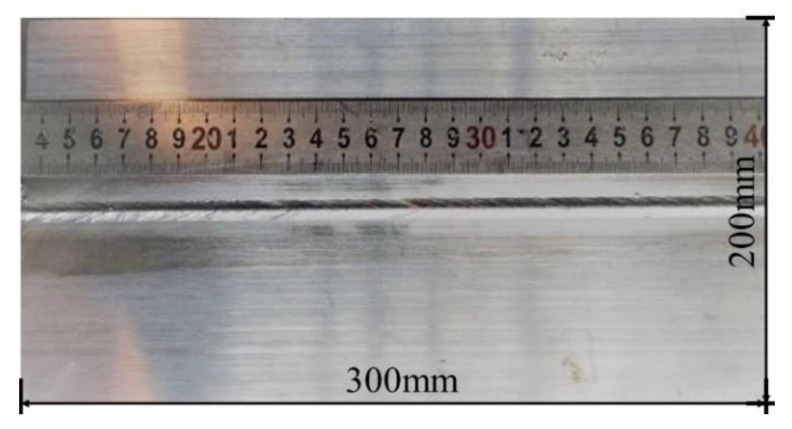
Macro morphology of weld with penetration of 23 mm.

The full penetration welding joint is obtained by the welding test, as shown in Figure 11, without obvious defects such as drop, sink, and biting edge. X-ray beam inspection produced by Huangshi Shangshang testing equipment Co., Ltd. in Shanghai, China, was used to inspect the welding joint, and no defects such as cracks or porosity were found inside.

**Figure 12:**
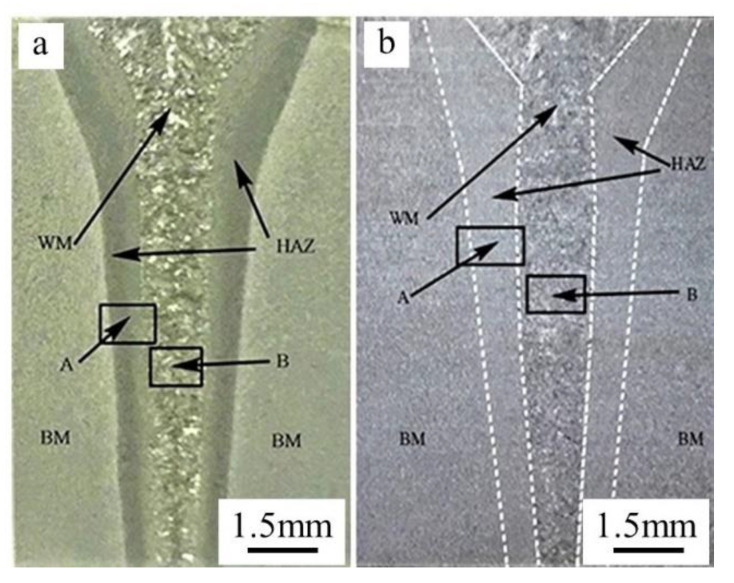
Macroscopic morphology of welded joint (**a**) before quenching and tempering treatment, and (**b**) after quenching and tempering treatment.

The cross section of EBW-joints shows an obvious “nail” shape with the maximum zone width of the heat-affected and welding seam up to 1.2 mm and 5 mm, respectively (Figure 12). Under welding conditions, it can be seen that the boundaries of the BM, the HAZ, and the weld zone are clear (Figure 12a), while after quenching and tempering, the weld structure is still clear, but the boundary between HAZ and BM is no longer clear (Figure 12b).

#### 3.3.2. Microstructure of HAZ

**Figure 13:**
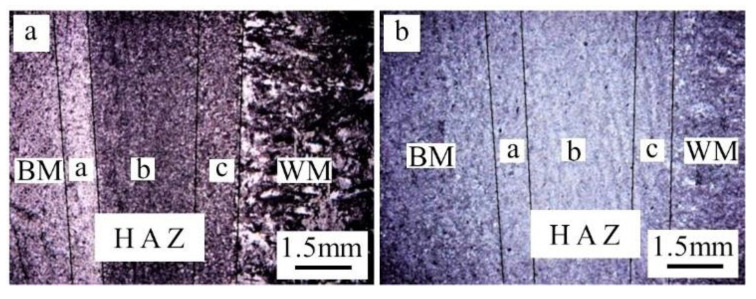
Metallographic structure of welded joints (amplification of Figure 12a) (**a**) before quenching and tempering treatment, and (**b**) after quenching and tempering.

Figure 13 represents the metallographic structure of the heat-affected zone before and after the quenching and tempering treatment. The heat-affected zone is distributed on both sides of the welding seam, and its maximum width is only 1.2 mm. It also consists of a complete quenching zone, incomplete quenching zone, and tempering zone.

On the fusion line, since the temperature is close to the melting point during welding and the temperature gradient is larger than the weld center, there is a lot of lath martensite in the fusion zone after rapid solidification (Figure 13a) [19]. At the same time, there is a large number of ferrite-forming elements in CLAM, and during the welding process, the δ ferrite formed at a high temperature remains at room temperature; therefore, there is a small amount of δ ferrite in the fusion line. The complete quenching zone is composed of a coarse-grain zone and fine-grain zone. The peak temperature in this zone is above A3 (1195 K) [16], which completely transforms into austenite structure in the welding process. In the coarse-grain zone, overheating, carbon content and alloy elements are the main factors affecting the grain size [20]. CLAM has low carbon content, so austenite grain grows obviously in the welding process, then forming lath martensite after rapid cooling. Afterword, it formed the fine-grain zone, due to the lower peak temperature and large temperature gradient in the fine-grain zone, the austenite grain size grew too late to become large, and thus formed tiny lath martensite. In an incomplete quenching zone, BM was heated to AC1 (1135K) ~ AC3 (1195 K) [16] temperature in the process of welding, the parent metal turned into fine austenite grain, then converted into tiny martensite in the fast cooling process after welding. As the temperature of the tempering zone is below AC1 in the welding process, tempered martensite remains.

After quenching and tempering, the δ ferrite in the whole HAZ is eliminated, and the organizations in this region returned to tempering martensite (Figure 13b).

#### 3.3.3. Microstructure of Welding Seam

**Figure 14:**
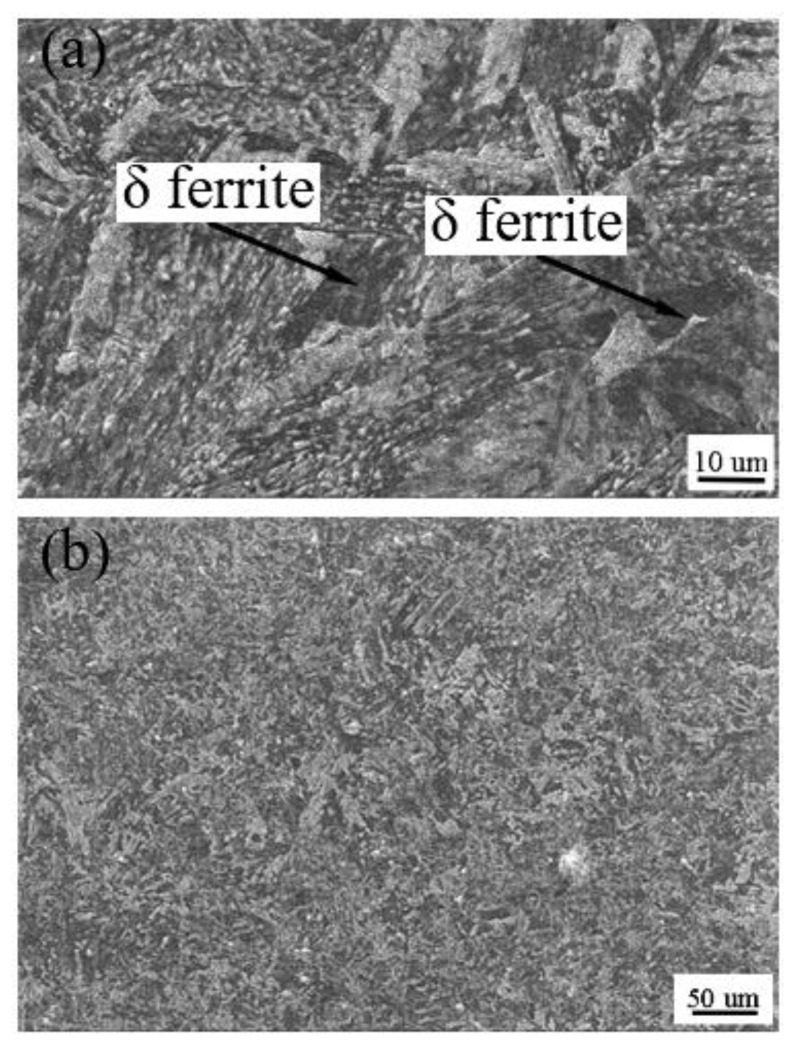
Microstructure of welding zone (amplification of Figure 13) (**a**) before quenching and tempering treatment (SEM), and (**b**) after quenching and tempering treatment (SEM).

Figure 14a also shows the microstructure of the welding seam. Due to the fast welding speed of the electron beam and high concentration of the energy density, there is a large temperature gradient vertical to the weld direction near the welding seam, which is conducive to the growth of columnar crystals. Therefore, the weld microstructure is mainly composed of columnar crystal perpendicular to the welding seam, and grows up to and near the center line of the weld. As the temperature gradient decreases, a super-cooled zone with a narrow width appears in the center of the weld, and the liquid metal spontaneously nucleates and grows up to equiaxed crystal. The weld microstructure is composed of lath martensite and a small amount of δ ferrite (Figure 14a). The main characteristics of martensite are high hardness and high strength. There are many reasons for this, except for the carbon content, strengthening of phase transformation, strengthening of solid solution, etc. [20], which have a great influence on it. Hu Jie et al. [5] found that the δ ferrite in the weld is mainly present in the form of polygons and needles. The formation of δ ferrite is mainly affected by two factors: the composition of the metal in the weld and the thermal cycling conditions of the weld. The content of ferrite forming elements in steel is relatively high; the contents of Cr and W are as high as 8.96% and 1.48%, respectively, which makes the austenite phase region shrink and the δ ferrite phase region expands. Under equilibrium cooling conditions, due to the high energy density and fast welding speed of EBW, the δ ferrite precipitated from the liquid phase through nucleation and growth mode cannot completely transform into austenite, so the weld still has the residual δ ferrite. At the same time, the austenite finally transforms into the coarse lath martensite structure due to the high heat input of the weld and the fast-cooling speed. The lath martensite is a low carbon supersaturated α-ferrite solid solution with high density dislocation, which has high brittleness. Moreover, due to the welding characteristics of electron beam, there is a great internal stress in the process of solid-state transformation of the weld, so the weld joints are obviously prominent after stretching (Figure 9c) and side bending (Figure 10a), and the weld impact energy is very low (as shown in Figure 9). Another reason for the low-impact energy is the coarse-grain caused by high energy injection during EBW. After quenching and tempering (Figure 14b), although some of the coarse lath martensites have not been refined completely because of their heredity, they have been transformed into tempered martensites. The comprehensive mechanical properties are improved, and the microstructure is similar to the BM. After tensile and lateral bending, the weld structure is no longer prominent, and the impact energy and hardness are close to the BM.

### 3.4. Hardness Analysis

**Figure 15:**
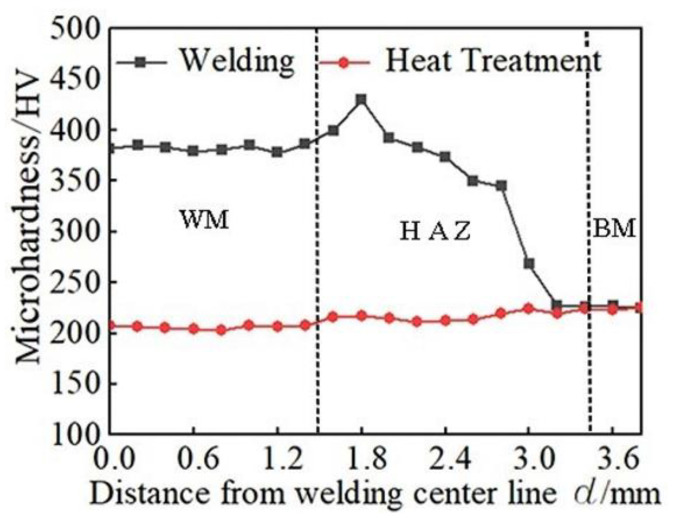
Hardness graph.

The microhardness distribution of welded joints is shown in Figure 15. Before quenching and tempering treatment, the hardness of all points in the welded joints area is greater than 225 HV, except the tempering area. As the distance from the weld center decreases to near the fusion line, the hardness gradually increases to the highest 430 HV, and then slightly decreases. The hardness gradually increases from tempering zone to the incomplete quenching zone due to the increase of martensite content in the structure. The martensitic structure in the fine grain area has a small grain size, which is the area with the highest hardness in the whole welding joints, and it is 430 HV. The hardness of the coarse-grain zone decreases slightly. Although the main structures are coarse lath martensite, the temperature gradient in the fusion line and coarse-grain zone are larger than the welding zone; these cause the hardening tendency of this area large, so the hardness is higher than the welding area. After quenching and tempering treatment, the microstructure of the welded joint recovers to tempered martensite, and the hardness of the fine grain area decreases the most obviously, mainly because the lath martensite in the fine grain area is small, which makes the carbide precipitate relatively easily during the quenching and tempering treatment, so the hardness of the joint decreases greatly. After tempering, the hardness of the whole structure is slightly lower than that of the BM.

## 4. Conclusions

The electron beam welding of 23-mm-thick CLAM steel plate has been realized. The weld forms well, and no defects were found inside it through X-ray beam inspection. The microstructure of joints is composed of coarse strip martensite and a small amount of δ ferrite. The complete quenching area is composed of a coarse crystal area and fine crystal area. Although the grain size is different, all the structure is lath martensite, and the incomplete quenching area is composed of small strip martensite with a small amount δ ferrite.

Under the influence of carbon content, transformation strengthening, and solution strengthening, the martensite has the characteristics of high hardness and high strength. At the same time, under the influence of embrittlement and hardness, there is hardening phenomena on welding joints before heat treatment, of which the fine grain zone has the highest hardness, and its hardness value is 430 HV. The room temperature impact toughness is poor, the maximum impact energy is only 19 MPa, and the residual stress is large as a whole.

After the quenching and tempering treatment, the δ ferrite structure was eliminated, and the welding seam changed into the same tempered martensite structure as the BM. After the quenching and tempering treatment, the impact toughness was greatly improved, and the hardness and residual stress decreased as a whole.

## Figures and Tables

**Table 1:** Chemical composition of CLAM steel (wt %).

**Cr**	**C**	**W**	**V**	**Ta**	**Mn**	**Si**
8.96	0.093	1.48	0.16	0.10	0.48	0.042
**O**	**N**	***p***	**S**	**Ti**	**Fe**	
<0.01	<0.02	<0.005	<0.005	<0.01	Bal.	

**Table 2:** Welding parameters.

Voltage U_a_/kV	Beam Current I_b_/mA	Focusing Current I_f_/mA	Velocity V/mm/s	Working Distance/mm	Oscillation Shape	Oscillation Frequency f_p_/Hz
150	50	2480	10	300	circular	500
150	45	2440	10	300	circular	500
150	45	2440	5	300	circular	500

## Data Availability

The data presented in this study are available on request from the corresponding author.
